# Inflammation and Microvasculopathy in Renal Ischemia Reperfusion Injury

**DOI:** 10.1155/2012/764154

**Published:** 2012-02-21

**Authors:** Daniel Patschan, S. Patschan, G. A. Müller

**Affiliations:** Abteilung für Nephrologie und Rheumatologie, Universitätsklinikum Göttingen, Robert-Koch-Straße 40, 37077 Göttingen, Germany

## Abstract

Acute renal failure (ARF) severely worsens prognosis of hospitalized patients. The most frequent cause of intrarenal ARF is transient or prolonged renal hypoperfusion (ischemia). Ischemia primarily affects the function and structure of tubular epithelial cells, which, in severe cases, is characterized by epithelial cell necrosis. Nevertheless, ischemia does not exclusively lead to alterations of epithelial cells but also causes interstitial inflammation and interstitial microvasculopathy. Both inflammation and microvasculopathy are particularly important in terms of postischemic kidney repair. Postischemic microvasculopathy is characterized by endothelial cell swelling with subsequent microvascular occlusion. Thus, reperfusion is inhibited (no-reflow phenomenon). Such endothelial cell dysfunction offers new therapeutic perspectives in ischemic ARF. Newer observations point towards the role of the so-called endothelial progenitor cells (EPCs) in the treatment of ARF. Systemic administration of EPCs to mice with bilateral renal ischemia mitigates postischemic endothelial cell dysfunction and protects animals from acute renal failure.

## 1. Introduction

**Figure 1:**
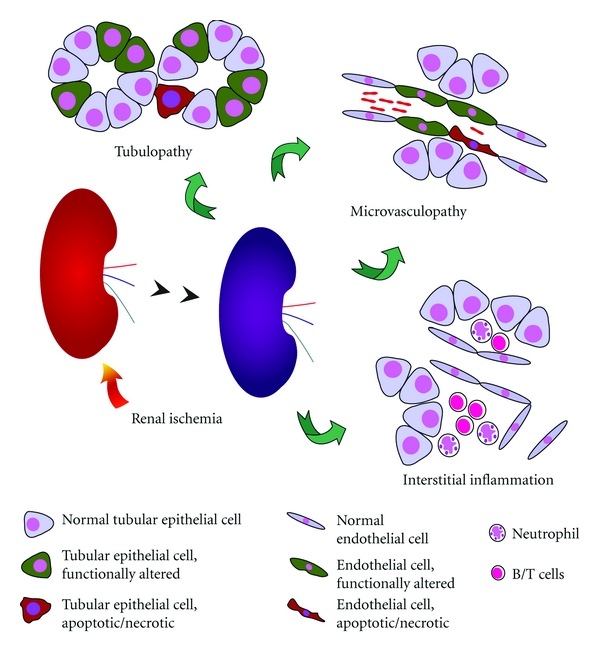
Acute ischemic renal failure is characterized by tubular epithelial cell dysfunction and damage. Postischemic restoration of kidney function and structure critically depends on both interstitial inflammation and peritubular microvasculopathy. The latter causes ongoing renal hypoperfusion even if ischemia has been corrected.

Acute renal failure (ARF) severely worsens prognosis of hospitalized patients. Approximately 1–5% of all patients treated in the hospital develop ARF [1]. The clinical significance of ARF results from its high mortality, which still today ranges from 30 to 70% [2]. ARF is categorized depending on its primary cause. Prerenal failure results from transient renal hypoperfusion. It is a functional response of a structurally intact kidney to hypoperfusion [3]. While postrenal ARF is caused by urinary tract obstruction with or without subsequent damage of renal tissue, intrinsic or intrarenal ARF is caused by diseases that either affect the glomeruli, the vasculature, the interstitium, or the tubules. The difference between prerenal and intrarenal failure due to hypoperfusion lies in the presence of structural tubular damage in the latter. The most frequent cause of intrarenal ARF in hospitalized patients is transient or prolonged renal hypoperfusion (ischemia reperfusion injury—IRI) [4–6]. Renal IRI is of particular importance in the setting of kidney transplantation [7]. Ischemia primarily affects the function and structure of tubular epithelial cells, which, in severe cases, is characterized by epithelial cell necrosis [8]. Nevertheless, IRI does not exclusively lead to alterations of epithelial cell function and structure but also causes interstitial inflammation and interstitial microvasculopathy (Figure 1). These alterations can delay restoration of renal function which potentially worsens prognosis of patients with ischemic ARF [3]. Postischemic microvasculopathy is characterized by endothelial cell swelling, leading to prolonged ischemia even if the primary cause of hypoperfusion has been eliminated [9, 10]. Such *no-reflow phenomenon* has also been described in other organs [11]. In recent years, it has become more and more evident that by targeting postischemic renal microvasculopathy, kidney function can partially or completely be preserved [9, 10]. First investigations were performed by the group of Goligorsky [12]. Immunoincompetent rats with renal IRI were injected with mature endothelial cells from humans (human umbilical vein endothelial cells—HUVECs). Animals were not only protected from ARF, but histological analysis showed direct incorporation of HUVECs into the endothelial cell layer within the renal microvasculature [12]. In subsequent years, comparable therapeutic effects were demonstrated for so-called endothelial progenitor cells (EPCs). In this paper, we will summarize the current knowledge on postischemic interstitial inflammation and microvasculopathy, and we will discuss therapeutic strategies to target microvasculopathy in acute ischemic renal failure.

## 2. Interstitial Inflammation

During the last 15 years, our knowledge of postischemic inflammation in the kidney has significantly been increased. The inflammatory process is initiated by both endothelial and tubular cell dysfunction. A number of different proinflammatory/immunomodulatory cytokines, such as IL-1, -6, and -8, TGF-*β*, TNF-*α*, and MCP-1 (monocyte chemoattractant protein-1), are released into the renal tissue and the circulation, respectively [13, 14]. Serum levels of IL-6 have been shown to indicate a higher risk of death in patients with acute kidney injury (AKI) [15]. Recently, Toll-like receptor 4 (TLR 4) has been shown to play an essential role in postischemic renal IL-6 production [16]. In this study, leukocytes from TLR 4 knockout mice (TLR 4 (−/−)) infiltrated kidneys of TLR 4-expressing animals (TLR 4 (+/+)), but there was almost no impairment of renal function. In addition, only leukocytes from TLR 4 (+/+) mice produced IL-6 in response to high-mobility group protein B1 (HMGB1). The renoprotective consequences of TLR 4 inactivation have also been documented by Zhang and colleagues [17]. Earlier studies showed that TLR 2 represents an important regulator of the proinflammatory response as well. Renal tubular epithelial cells displayed increased TLR 2 expression after acute ischemia, and TLR 2 inactivation protected mice from AKI [18]. TLR 2 (−/−) mice did not only show decreased intrarenal expression of MCP-1, TNF-*α*, IL-6, and IL-1, but tissue infiltration by neutrophils was also markedly reduced.

Postischemic tissue infiltration by certain populations of inflammatory cells is a hallmark in renal IRI. Neutrophils, macrophages, natural killer cells, and different subtypes of T cells home to the interstitial space where they modulate the inflammatory response [3, 19–24]. The earliest population of cells that accumulate within peritubular capillaries, the interstitium, and to some extent within tubules are neutrophils [3]. Nevertheless, the clinical significance of neutrophil infiltration can be doubted or at least remains a matter of debate, although blockade of neutrophil function partially protects animals from AKI in some models [20]. Depletion of macrophages also ameliorates renal function [21] in ischemic AKI. However, macrophage activity critically depends on the function of T cells. The data on the role of T cells in renal IRI are conflicting. Nu/nu-mice, which neither produce CD4^+^ nor CD8^+^ T cells, displayed higher ischemia tolerance as compared to wild-type animals [23]. Selective reconstitution of CD4^+^ T cells significantly worsened renal function after ischemia. Comparable renoprotective effects were observed in Rag-1 knockout (KO) mice. Such animals have small lymphoid organs that do not contain mature B and T cells [25]. The recombination activating genes-1 and -2 are required for somatic assembly of both the B- and T-cell receptors [26]. Ischemia protection in Rag-1 KO mice was reversed if the animals were adoptively transferred with WT CD4^+^ cells. These effects critically depended on the cells' ability to produce Interferon-*γ* [24]. Nevertheless, it is doubtable that CD4^+^ T cells exclusively mediate deleterious effects on renal function. Recently, Lee and colleagues investigated the role of CD4^+^/CD25^+^ (regulatory) T cells in cisplatin-induced AKI [27]. Nu/nu-mice showed increased survival, reduced tubular epithelial cell damage, and decreased tissue levels of TNF-*α* after administration of CD4^+^/CD25^+^ T cells. Comparable data were published by Yokota and colleagues [28]. Depending on their respective phenotype, CD4^+^ T cells were shown to either act protective or deleterious in the setting of IRI. Such modulatory actions partly depended on the balance between INF-*γ* and IL-4 production [28]. In summary, the role of T cells in AKI is rather complex, and it can be assumed that individual biological properties of the different subsets of T-cells are fundamentally important in the process of kidney repair after ischemia. While T cell-mediated effects on postischemic kidney repair and function seem to require the presence of the cells in the kidney, this is not mandatory with B cells. B-cell depletion has been shown to partly protect from ischemia-induced structural damage, although neutrophil and T-cell infiltrates were not diminished [29]. Interestingly, susceptibility to ischemia could be reestablished by serum transfer from wild-type animals. Transfer of B cells in contrast was not associated with decreased ischemia tolerance. Whether these effects were exclusively related to antibodies remains unclear [29].

Natural killer T-cells (NKT cells) belong to the T cell family of lymphocytes. They express cell surface marker molecules of conventional T cells, but in addition they are positive for NK1.1, also known as NKR-P1.9 (natural killer cell receptor P1.9) [30, 31]. The T-cell receptor of NKT cells recognizes glycolipids presented by the class I-like molecule CD1d [30]. NKT cells can produce a diverse group of cytokines in response to antigen recognition which amplifies the activity of dendritic cells, conventional T cells, regulatory T cells, other NKT cells, and B cells, respectively [30]. In an elegant study, Li and colleagues [32] investigated the role of NKT cells in murine IRI. Renal ischemia of 30 minutes was followed by NKT cell and neutrophil accumulation in the kidney and by significantly increased IFN-*γ* tissue levels. Inhibition of NKT cell activity by cell depletion decreased numbers of IFN-producing neutrophils in the kidney and protected mice from AKI [32].

Another hallmark of IRI-associated inflammation is activation of the complement system [3, 33]. The complement cascade is represented by more than 30 plasma proteins which predominantly are produced by the liver. They are critically involved in the innate immune response. Complement activation can occur by binding of C1q to the Fc fragment of antibodies (classical pathway) and, on the other hand, the cascade is permanently activated by spontaneous degradation of complement factor C3 (alternative pathway) [34]. Factor C5a has been shown to play an important role in IRI-induced kidney inflammation [33]. As a matter of fact, the C5a receptor is expressed by tubular epithelial cells and by certain interstitial macrophages, respectively [35]. IRI increases C5a receptor expression in the proximal tubule [36]. C5a acts as potent chemoattractant which results in the recruitment of neutrophils, monocytes, and T cells [3]. Administration of anti C5a mAB has been shown to block neutrophil and macrophage invasion after renal ischemia and to protect mice from renal dysfunction [36]. Although complement activation in murine IRI is predominantly mediated by the alternative pathway [37], a recent study identified the classical pathway to be the essential complement activator in human IRI [38]. Hypoxia treatment of proximal tubular epithelial cells from humans (PTEC) induced significant complement activation. By using C1q-depleted serum or by blocking C1q with antibodies, the classical pathway was shown to be critically responsible for initiating the cascade. Furthermore, this process was mediated by IgM which binds to PTEC in response to hypoxia [38].

The consequences of interstitial inflammation for the treatment of renal IRI are currently investigated. IL-10, which acts as an anti-inflammatory mediator, protected against ischemic AKI by inhibiting Th1 cell function [39]. Comparable renoprotective effects were observed with the administration of anti-IL-6 mAb. This approach significantly inhibited production of proinflammatory mediators and tissue infiltration by neutrophils [40]. Very interesting results are related to effects of *α*-melanocyte-stimulating hormone or melatonin. The hormone, also known as N-acetyl-5-methoxytryptamine, is produced by the pineal gland, and it is released into the circulation in a circadanic manner [41]. It had once been discussed to act as key regulator in sleep-wake rhythm [42]. Although this concept has been modified in recent years, the hormone has been shown to be involved in a number of other physiological events, namely, the detoxification of free radicals [9]. In addition, melatonin can inhibit activation of proinflammatory genes which cause renal injury after ischemia. In this setting, it even augments anti-ischemic actions of erythropoietin and protects mice from AKI-associated lung injury [43].

If such strategies will be established in human AKI one day remains speculative at the moment, but one has to be aware of the fact that renal IRI is neither an exclusive tubular nor vascular disease but also a severe inflammatory process. Postischemic renal inflammation may also contribute to microvasculopathy in IRI, which will be the topic of the next section.

## 3. Microvasculopathy in Renal IRI

Microvasculopathy significantly contributes to ongoing postischemic kidney dysfunction. Despite the fact that renal hypoperfusion mainly causes functional and structural alterations of the tubular epithelium, studies performed in recent years pointed toward the role of postischemic endothelial cell dysfunction (ED) in peritubular capillaries as an important perpetuating factor of prolonged kidney malfunction [12, 44].

First evidence for the role of postischemic ED in acute ischemic kidney injury came from studies performed in the early 1970s [45]. Rats undergoing renal artery clamping displayed swelling of all cellular elements in the kidney which caused persistent renal hypoperfusion even after reperfusion. Swelling of endothelial cells significantly contributed to such *no reflow*. Extracellular fluid expansion, induced by hypertonic mannitol solution, partly prevented swelling of endothelial cells and thus protected from (post)ischemic renal damage. Hence, cell swelling had been identified as pathogenetic factor in tissue ischemia [46]. As a matter of fact, postischemic ED has to be considered as global cellular dysfunction syndrome, characterized, in addition to cell swelling, by increased paracellular and transcellular endothelial permeability and by increased endothelial expression of different types of cell adhesion molecules. Among those are P- and E-selectin and ICAM-1, respectively. The two selectins and ICAM-1 mediate leukocyte-endothelial interactions, a prerequisite for transvascular leukocyte migration. Inhibition of the selectins and of ICAM-1 has been shown to reduce renal injury in IRI [47]. Postischemic ED does not exclusively perpetuate acute kidney dysfunction but also worsens long-term outcome of renal IRI [48]. Studies performed in 2001 showed permanent damage of peritubular capillaries after acute renal ischemia [49].

Taken together, these observations pointed toward a new therapeutic approach in ischemic AKI: targeting of postischemic ED [50]. First evidence for the viability of such treatment came from studies performed by the group of Goligorsky [12, 44]. Systemic injections of mature endothelial cells from humans (HUVECs—human umbilical vein endothelial cells) into immunoincompetent nude rats protected the animals from ischemic kidney damage. *In vivo* microscopic analysis showed postischemic endothelial cell swelling within the peritubular capillary network, and, in addition, showed that complete normalization of microvascular tissue perfusion occurred as late as 24 hours after ischemia. In this setting, systemic administration of HUVECs markedly inhibited swelling of endothelial cells and promoted a faster functional and structural recovery of the organ. Histologically, injected cells had partly been incorporated into the endothelial layer of small blood vessels surrounding the tubular integrity. These studies showed for the first time that targeting of postischemic ED by the administration of cells of the endothelial lineage is a true option in the treatment of acute ischemic kidney injury. Although such therapeutic approach is promising, a number of questions remain. The most relevant question is related to the source of cells of endothelial origin. If endothelial-type cells are supposed to be administered in acute kidney injury, they must become available within a short period of time. The next problem is related to the immunological acceptance of exogenously injected cells. In an optimal setting, cells would rapidly be isolated from the recipient in order to become available for immediate systemic administration if necessary. A possible alternative may be represented by the so-called endothelial progenitor cells (EPCs) which will be reviewed in the last section.

## 4. Endothelial Progenitor Cells (EPCs) in Renal IRI

EPCs were described for the first time in 1997 [51]. Cells expressing CD34 were isolated from human umbilical vein blood and, after several days of culturing, systemically injected into immunoincompetent animals with ischemic lesions of the lower extremities. This measure did not only improve postischemic blood flow, but microscopic analysis showed direct incorporation of injected cells into the endothelial layer of blood vessels within the reperfused tissue. For many years, it has been assumed that EPCs are more or less exclusively derived from pluripotent hematopoietic stem cells in the bone marrow. Meanwhile, this concept has significantly been modified. According to the current literature on EPC biology at least two major populations of EPCs have to be differentiated [52–55]. The first and by far more in detail analyzed population is represented by so-called *endothelial cell-like cells *(EC-like cells) or *early endothelial outgrowth cells* (eEOCs) [9]. Early endothelial outgrowth cells develop from hematopoietic stem cells in the bone marrow and they express both, endothelial and hematopoietic cell marker molecules [55]. In addition, they are capable of differentiating into cells of the hematopoietic lineage. Culturing eEOCs from mononuclear blood cells takes 5–7 days and it has reproducibly been shown that the cells can act anti-ischemic in different experimental situations. A number of studies evaluated the diagnostic and therapeutic value of eEOCs in ischemic heart disease [56–58]. Although initial studies by Asahara et al. [51] suggested that EPC-mediated vascular repair results from direct cell incorporation into the endothelial layer of small blood vessels, newer concepts favor indirect mechanisms to be responsible for vasoprotection. Thus, EPCs home into the postischemic tissue where they release different proangiogenic mediators such as VEGF (vascular endothelial growth factor), HGF (hepatocyte growth factor), and IGF-1 (insulin-like growth factor-1), respectively [59]. These substances promote a faster recovery of damaged endothelial cells [9, 60]. The second EPC-subpopulation is represented by the so-called *late endothelial outgrowth cells* (lEOCs) or *endothelial colony-forming cells* (ECFCs) [54, 55]. Late outgrowth endothelial cells can also be cultured from mononuclear blood cells, but in contrast to eEOCs, they are not capable to differentiate into cells of the hematopoietic lineage [9]. *In vitro* studies showed significantly stronger formation of vessel-like structures with lEOCs than with eEOCs. In a newer review paper by Yoder and Ingram [54], it has even been questioned if lEOCs are true *progenitors* of endothelial cells since they share a number of characteristics with mature endothelial cells. The only difference between mature endothelial cells and lEOCs lies in the higher proliferative potential of the latter. Nevertheless, proangiogenic or anti-ischemic effects have been shown for both cell types, eEOCs and lEOCs. The vast majority of studies performed over the last 14 years investigated the role of eEOCs.

Acute ischemic renal failure is characterized by severe endothelial dysfunction [10, 12]. With regard to the promising studies by Brodsky et al. [12], the role of eEOCs in the treatment of acute ischemic renal failure was investigated for the first time about 6 years ago [61]. Early endothelial outgrowth cells were significantly mobilized by acute kidney ischemia and ischemic preconditioning of the animals induced eEOC homing into the postischemic tissue. Mononuclear cells isolated from such kidneys protected recipient animals from renal failure which was the proof-of-principle that eEOCs can serve as therapeutic tool in iAKI. Meanwhile, different strategies to increase renoprotective effects of eEOCs in iAKI have been established. In 2009, the substance 8-pCPT-2′-O-Me-cAMP (Epac-1 Ac) has been shown to augment the efficiency of an eEOC-based therapeutic regimen in iAKI [62]. Epac-1 Ac induced redistribution of *β*1-integrins towards the plasma membrane of eEOCs which increased eEOC homing into the renal tissue. Newer and yet unpublished data indicate significantly eEOC stimulating effects of the hormone melatonin. Melatonin-pretreated eEOCs showed higher renoprotective potential than untreated cells. The responsible mechanisms were not related to increased homing of the cells, but melatonin acted antiapoptotic, stimulated VEGF secretion by the cells, and increased migration of eEOCs *in vitro*. In addition, supernatant from melatonin-treated eEOCs induced faster migration of mature endothelial cells.

Together, these data clearly show that eEOCs are a reliable option to ameliorate the short-term prognosis of iAKI. Current own investigations focus on the process of fibrogenesis after acute kidney ischemia. The mid- to long-term prognosis of iAKI critically depends on the amount of postischemic renal fibrosis [63]. It has recently been shown that EPCs inhibit renal fibrogenesis in two models of chronic renal failure [64, 65]. It seems most likely that eEOCs can be employed as antifibrotic therapeutic tool in iAKI and as a matter of fact, first own and also yet unpublished observations seem to confirm this theory.

Nevertheless, a number of questions still need to be answered before eEOCs will finally become usable for treating patients with iAKI. The most significant problem is still related to the time frame which is needed for the cells to be isolated. Further investigations will have to be performed in order to optimize the process of cell enrichment for therapeutic administration.

## 5. Conclusion

Acute *ischemic* renal failure (iARF) is the most frequent type of acute renal failure in hospitalized patients. Although renal hypoperfusion primarily affects the function and structure of the tubular epithelium, alterations of the microvasculature and inflammatory processes within the interstitial space are of particular importance with regard to postischemic restoration of kidney function. Postischemic microvasculopathy, which in severe cases is characterized by obstruction of the peritubular vasculature, can potentially serve as therapeutic target in acute ischemic renal failure. Early endothelial outgrowth cells (eEOCs) are potent inhibitors of postischemic microvasculopathy in murine iARF, and systemic administration of the cells protects mice from acute renal failure after ischemia.
